# Design guidelines for animated data visualization based on perceptual capacity limits

**DOI:** 10.1186/s41235-026-00724-y

**Published:** 2026-03-31

**Authors:** Ouxun Jiang, Camillia Matuk, Madhumitha Gopalakrishnan, Wen Xu, Jason Dykes, Anastasia Bezerianos, Fanny Chevalier, Petra Isenberg, Steven Franconeri

**Affiliations:** 1 Northwestern University, Evanston, USA; 2 New York University, New York, USA; 3 Northeastern University, Boston, USA; 4 City St George’s, University of London, London, UK; 5 Université Paris-Saclay, CNRS, Inria, LISN, Orsay, France; 6 University of Toronto, Toronto, Canada

**Keywords:** Perception, Attention, Data visualization, Design

## Abstract

Data visualizations are used widely to help people see patterns in data across research, policy, education, and business. Computer screens allow these visualizations to become animated, which can effectively show processes of change. While animations can be engaging, ineffective design can also make them confusing or overwhelming. We develop new guidelines for designing effective animated data visualizations by reviewing 40 real-world visualization examples, and categorizing the visual tasks people perform when viewing them. These categories include tracking tasks, holistic judgments, and noticing objects added to or removed from a display. We then evaluate the known capacity limits of each task from human motion processing literature and use these to inform design techniques that enable visualizations to respect these capacity limits. Together, the tasks, limits, and corresponding techniques form new, broadly applicable guidelines that should help designers create effective animated visualizations informed by theory of human perception.

## Significance statement

Animated data visualizations are widely used to communicate changes in data. While they are engaging, they are also often confusing, because dynamic displays tend to overwhelm visual processing capacity limits. There are valuable existing guidelines for designing effective animated visualizations, but those guidelines are fragmented and offer insufficient explanation for why some animations remain manageable, while others tend to overwhelm. Our work addresses this gap by creating a framework of visual tasks that dynamic visualizations tend to tap, categorizing them as visually powerful or capacity limited, and showing how designers can make small changes that can lead to far better performance among viewers.

## Introduction

**Fig. 1:**
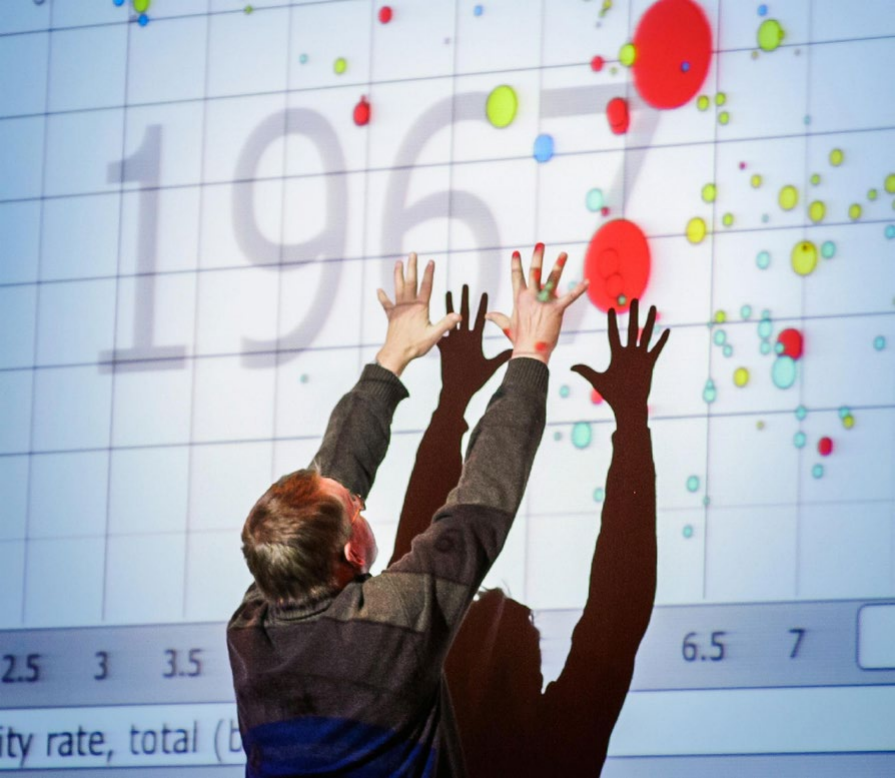
TED talk by Hans Rosling. *Note*. In his TED talk, Hans Rosling presented a rich animated data visualization and helped the audience interpret the patterns with his gestures and narrative. Later studies showed that without his guidance, viewers’ understanding of the animation was worse than when the same information was presented in a static graph (Fisher, 2010)

When well designed, visualizations can be powerful tools for exploration and explanation (Franconeri et al., 2021). Across educational settings, journalism, and software interfaces, animated visualizations are often used to show steps in a changing process, such as simulating the spread of an infectious disease in vaccinated versus unvaccinated populations (Harris et al., 2015). Journalists at outlets like *The New York Times Upshot* rely on animated visualizations to reflect changes in data over time (e.g., Badger et al., 2018; Cox, 2014). In a popular TED talk (Fig. 1), Hans Rosling, who is known for promoting the use of data to understand global issues, presented a rich bubble chart in which multiple data dimensions were represented using the X-axis, Y-axis, size, and color (Rosling, 2006). By augmenting this chart with animation, he showed how these variables changed over time, successfully telling a data story about how globalization has improved the health and wealth of poorer countries.

This case study shows that animation can be an effective way to depict change, such as how data values evolve over time, how a process unfolds step by step, or how the same data can be categorized differently. Consider the operation of a machine—while static visualizations can only show selected steps of the process (sometimes augmented with annotations, such as arrows, to convey movement), an animated version can better capture the movement of that machine in the real world (Tversky et al., 2002). Animations also allow viewers to see the intermediate steps and transitions rather than only the results or summaries of changes (Fisher, 2010). People can also more easily understand that two graphs present the same set of data, but ordered in different ways, when the points in the first graph are animated to move to their positions in the second graph (Heer & Robertson, 2007). Similarly, when detecting the appearance or disappearance of nodes and paths on maps, viewers have been shown to perform better with animated maps than with static frames, because the animated maps left visual traces of those changes (Boyandin et al., 2012). Viewers also reported preference for animations, finding them easier for noticing changes. This preference for animation over static alternatives is shown in other studies as well (e.g., Robertson et al., 2008), and many studies show that people feel more confident about learning data patterns from animations. For example, when university students learned about mathematics through animated videos, objective pre-tests and post-tests showed measurable improvement, but their subjective reports on visual imagery, information processing, and attention suggested that they generally overestimated the effectiveness of learning (Bos & Wigmans, 2025).

Animated visualizations can be engaging, but at the same time confusing. In fact, in many cases animations are often less effective, or at least no more effective, than static visualizations (Fisher, 2010; Hegarty et al., 2009). For example, elementary school students learning Newton’s Laws with an animation performed no better than those using a static equivalent on factual and application questions (Rieber, 1989). When answering questions about graphed trends (e.g., identifying the data subset with the largest increase), people took more time and showed lower accuracy with an animated graph compared to a static one (Robertson et al., 2008). Moreover, while animations often give viewers greater confidence in their learning, their objective performance typically suggests overestimation of comprehension, as the animated materials merely felt easier to perceive (Paik & Schraw, 2013).

Some studies report advantages for animation, but these studies often give the animated visualization an unfair advantage of including more information than the static counterpart or allowing more time to display the available information (Tversky et al., 2002). For example, one study showed that learning a step-by-step process (e.g., the functioning of a human heart) through an animation led to better learning outcomes on tests of drawing, terminology, and comprehension than learning through a static visualization. In this example, however, participants also spent more time studying the animated visualization than the static alternative. When viewing time was controlled, the advantage of learning from animation became less significant, so we cannot conclude that animation, rather than increased viewing time, is the only factor responsible for performance improvement. In general, when the information and the procedures in an animated visualization and a static visualization were made comparable, learning outcomes are typically the same, or animation is proved less effective than static equivalents (Tversky et al., 2002). While animation does not appear to offer better understanding in such time-matched comparisons, the fact that animations tend to be rated as more engaging might lead viewers to spend more time learning from them, which in turn could lead to learning advantages.

A major reason animations can be less effective is that they tend to overwhelm and confuse viewers by moving too many objects at once, often too quickly. People can generally track only three to four objects across an animation, unless objects move at a very slow speed and remain well spaced (Alvarez & Franconeri, 2007). Animations also tend to move too quickly and finish too quickly for viewers to have sufficient time to process the pattern of change (Sweller et al., 2011). In a comparison of different visualization types (e.g., animation and small multiples), animation yielded lower accuracy on some tasks, such as identifying specific directions, possibly because there were many fleeting frames involved and viewers had to remember quickly vanishing past frames (Peña-Araya et al., 2020). By contrast, static visualizations allow for repeated inspection of details over time. Re-inspection of the animation can mitigate this to some extent but can only be implemented by replaying the animation which can still result in a fleeting reception of information, and repeated replays may even increase the time required for understanding (Brehmer et al., 2020; Harrower, 2002; Tversky et al., 2002).

Empirically established guidelines can help designers create animated visualizations that are more understandable (Harrower, 2003; Li et al., 2025; Zheng et al., 2018), including slowing objects down, showing only a small number of changes at once, and allowing users to control the animation (Fisher, 2010; Tversky et al., 2002). But this list is limited, and there are many instances where these guidelines cannot explain why one visualization is perceptually manageable while another is not.

The research literature on motion perception provides a potentially fruitful wealth of knowledge about what kinds of motion people can easily process versus what tends to overwhelm them (e.g., Watamaniuk & Duchon, 1992; Watamaniuk et al., 1989). However, this knowledge has not been consolidated into a framework that connects motion perception research with the typical displays used in animated visualization designs. We propose to address this gap through a systematic approach, organizing the existing evidence to provide quantitative capacity estimates for well-defined perceptual tasks, such as: “*what is the maximum number of objects viewers can track simultaneously?*”.

Our goal is to generate research-backed design guidelines that enable practitioners to make conscious choices about their design strategies. Considering the perceptual processes involved in viewing animated visualizations, we first defined the *perceptual tasks* people commonly perform when viewing animated data visualizations. We did so by reviewing a wide and representative set of real-world examples of animated visualizations to create a taxonomy of these tasks. This yielded a set of common visual operations for perceiving animated visualizations, such as tracking paths of objects or seeing global patterns, along with the capacity limits associated with each task. Because these perceptual tasks can be loosely associated with communicative concepts that designers often aim to convey, we also mapped the tasks to the concepts drawn from an existing taxonomy of animation roles in interfaces (Chevalier et al., 2016). Finally, through our review of real-world examples and literature, we compiled a set of techniques to help designers avoid exceeding viewers’ perceptual limits, helping to ensure that animations effectively convey their intended patterns.

## Methods

To catalog common perceptual visual tasks as the basis of our guidelines, we drew on task lists from existing literature, including low-level analysis tasks describing people’s activities when using visualization tools to understand data (Amar et al., 2005), holistic coding types in data visualizations (Szafir et al., 2016), and animated transitions in statistical graphics (Heer & Robertson, 2007). We also reviewed research on human motion perception and visual cognition, such as multiple object tracking (MOT) and multiple identity tracking (MIT), to learn about current understandings of the capacity limits of perceptual tasks.

In addition, we collected and reviewed 40 real-world examples of animated data visualization to examine the visual tasks that we identify as what designers expect viewers to perform, as explained by the explanatory text that accompanied each animation. The examples are mostly from sources that are recognized for producing effective visualizations, either because they were created by externally recognized design professionals, had broad public reach and acclaim, or were used in formal learning environments. We also included examples from sources such as open forums where designers of varying skill levels share their visualization project, allowing us to capture both effective designs and those that could inspire redesign. To acquire a representative sample of animated visualizations, we identified sources drawing on our own team’s expertise in data visualization research, practice, and teaching (over twenty years of experience), consulting data visualization experts outside of our team for their trusted sources such as online news sites, and examining popular websites found through internet searches. Ultimately, our sources included data journalism sites (e.g., *The New York Times*) to reflect professional work, data visualization forums (e.g., the Reddit channel *dataisbeautiful,* https://www.reddit.com/r/dataisbeautiful/) to see work by a wider range of designers, and web-based environments featuring animated visualizations (e.g., NetLogo, Wilensky, 1999). For journalism sites and forums, we sorted posts by recency and selected those that: (a) showed real data; (b) employed animation as a primary function for showing data; and (c) featured visualization types (e.g., bar graphs, scatterplots, line graphs, etc.) that contributed variety to our catalog. While this sample selected by our current approach is not exhaustive, we consider it representative of the diversity of animated visualizations available.

With the goal of summarizing which visual tasks viewers are expected to perform, for each animation, one author and a research assistant assumed the role of the viewer and documented observations. Our notes include descriptions of which elements were animated (e.g., X-axis, bars), how they were animated (e.g., dots smoothly moving simultaneously, or data points added into the display one by one), the likely intended function of the animation (e.g., regrouping data, showing changes in trends over time), and other features such as scenario, number of moving objects, and motion speed. The descriptions were based on both the visualizations themselves and the accompanying text information provided by the authors. All information was compiled into a spreadsheet and later analyzed to consolidate a taxonomy of perceptual tasks by categorizing the examples and gradually synthesizing their intended tasks according to prior taxonomies (see Sect. “Visual Tasks and Design Techniques to Overcome Perceptual Limits”). To refine our taxonomy iteratively, we had feedback rounds with members of our team of collaborators who are experts across fields of perceptual psychology and data visualization. Based on our analysis of examples and review of previous work (e.g., Fisher, 2010; Mayer & Moreno, 2003), we also summarized techniques that designers can use to overcome the capacity limits of the perceptual tasks, including showing history traces, highlighting relevant objects, moving objects in stages, and incorporating interactivity. We then collectively and iteratively mapped these techniques to the perceptual tasks and provided suggestions for their application for each task. Finally, we consolidated the perceptual tasks and the techniques into comprehensive guidelines for visualization designers, which we present in an interactive webpage (current link). Based on our rigorous process, the resulting categorizations of tasks and techniques represent a well-informed hypothesis for making predictions about why some animations fail and how to fix them. In Discussion and Future Work, we point out how future empirical testing of those predictions will be important for validating the usefulness of these taxonomies.

## Visual tasks and design techniques to overcome perceptual limits

To better understand how people process animated data visualizations, we created a taxonomy of the perceptual tasks by consolidating existing taxonomies from visualization and motion perception literature. Each task has a capacity limit on how much of the motion can be processed at once. We organize these perceptual tasks into three major categories: (1) tracking tasks (tracking objects as a set, tracking objects as different individuals with identities, or tracking certain features), (2) holistic judgments (extracting average features and evaluating changes to a shape envelope), and (3) noticing objects being added or removed. For each task, we also provided examples of associated concepts that designers tend to communicate (Chevalier et al., 2016) when requiring each task—for example, making holistic judgments is typically associated with seeing changes to the overall data pattern.

We identified and summarized techniques that could help designers overcome perceptual limits of the listed visual tasks. The list of techniques was drawn from our analyses of real-world animated data visualizations and cross-referenced with findings from visual perception literature. Below, we include a discussion of suggested design techniques after introducing each visual task. While no review can cover all possible techniques, we are confident that many of the most powerful design guidelines are represented.

### Tracking tasks

**Fig. 2:**
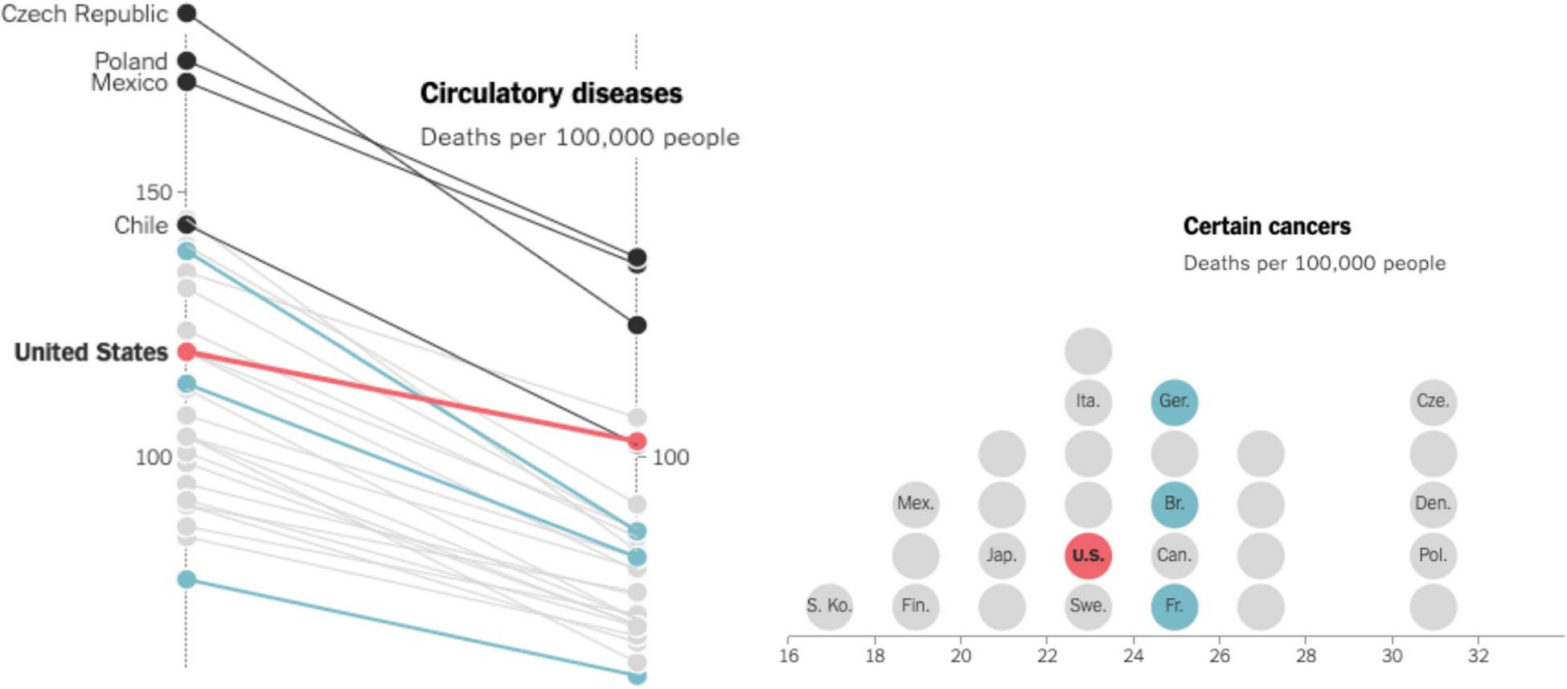
Example of tracking in animated data visualization. *Note*. Example from *The New York Times* (Buchanan et al., 2014). The parallel coordinates plot on the left, showing circulatory diseases in several countries across years, transitions into the dot plot on the right, showing deaths from certain cancers in the same countries. During this transition, the chart on the right reflects data from the same timepoint as in the right vertical axis in the chart on the left. This example expects the viewer to track where the highlighted dots ended up after the transition, and the designers visually differentiated these dots with a consistent and distinguishable color encoding during the transition to make tracking more manageable

Tracking tasks occur when designers expect viewers to track positional changes occurring in a specific set of graphical objects, i.e., to *monitor data value changes of some data objects*. Tracking tasks also involve viewers being expected to *track data objects during an axis change*. During an animation, the axes of the visualization may change in the variables represented on the axes, the range of values on the axes, or the orientation of the axes. Tracking enables viewers to understand how objects before and after such changes to the axes correspond to each other. For example, in Fig. 2, the parallel coordinates plot (left) changes into the dot plot on the right, where the vertical axis on the left (deaths from circulatory diseases) changes to the horizontal axis on the right (deaths from certain cancers). By tracking selected dots (countries) as they move, viewers can see how these dots correspond across the two visualizations and how the two disease variables relate to each other for the presented countries.

We identified three types of tracking tasks people perform:

#### Tracking objects (or clusters) as a set (limit 3–4 objects/clusters)

When people track the positions of a subset of moving objects, they are typically limited to tracking around four at once, and this limit can drop even further in crowded displays or at higher speeds. These results come largely from perceptual psychology studies where participants track a subset of moving target objects within visually identical moving distractors (Pylyshyn & Storm, 1988). In a typical tracking task, around 6–12 visually identical objects (e.g., circles) are presented and a few are designated as targets by briefly flashing or changing color. After the objects move around the screen, viewers report if a probed object is one of the targets. Early work suggested a consistent capacity of tracking four targets (Pylyshyn, 2004).

Tracking performance degrades when the targets move closer to each other or to distractors, or when targets are within the same visual hemisphere (for review, see Scimeca & Franconeri, 2015). Crowding of targets attracts the eyes and increases visual acuity to avoid losing track of the targets (Hyönä et al., 2019). Performance can also be reduced by high moving speeds of the objects (distance over time). This effect may be driven by the speed itself (Holcombe & Chen, 2012) though competing accounts suggest that this impairment is not due to the higher speed per se, but to the increased number of potential target-distractor confusion during close interactions, even when the number of total objects stays the same (Franconeri et al., 2010). For design purposes, the impact is the same, such that higher overall speed of the objects impairs viewers’ ability to track.

While tracking is limited to 3–4 individual objects, that limit can extend to 3–4 groups of objects that are connected by some types of visual grouping cues. For example, tracking sets of objects can be cued by a bundling technique, where a set of objects with spatial proximity and similar moving directions are aggregated into one group to follow a shared path, instead of moving as scattered objects, allowing viewers to track more objects (Du et al., 2015; but see Levinthal & Franconeri, 2011).

#### Tracking objects (or clusters) as individuals, while remembering their individual features (limit 1–2 objects/clusters)

When treating objects as a single set, people can typically track 3–4 objects. But when they additionally need to differentiate the identity of each tracked object, capacity drops to 1–2 objects (Cohen et al., 2011; Horowitz et al., 2007; Shooner et al., 2010). For example, imagine an animated dot plot where each dot represents one country, viewers may need to remember which dot corresponds to which country if labels of the country names are only revealed when hovering over the dot with the mouse pointer. Because the dots are otherwise visually identical, this task imposes strong tracking demands. The same low capacity of 1–2 objects appears when viewers are asked to track objects while remembering the individual history of tracked objects, such as knowing whether the objects changed color, size, or trajectory (Saiki, 2002).

The attempt to track identities competes substantially with an attempt to track multiple object locations. When participants were instructed to focus on tracking object locations, location-tracking performance improved but identity-tracking performance decreased even when object identities remained visible until the test phase (Cohen et al., 2011; Oksama & Hyönä, 2008). One suggested explanation is that a viewer must continually remap the object identity to an object’s new location as it moves or that mapping could be lost. One likely mechanism is to move the focus of attention among the objects serially, continuously updating a link between identity and position at each step. Eye-tracking evidence supports this account that viewers actively moved their eyes among tracked objects when they are required to remember their identities, compared to when they tracked those objects as a single set. These eye movements become more frequent for faster-moving objects, suggesting a need to remap their identities even faster (Oksama & Hyönä, 2016).

Due to the low capacity of this task, viewers are unlikely to spontaneously track the identities of a large number of objects. Designers should therefore be aware of the potential challenges and use design strategies to facilitate this task, which we describe later in this paper.

#### Tracking objects by their features (1–2 features)

In some cases, viewers can track objects based on shared features, such as a similar color, shape, size, or direction of motion. In a scatterplot with dots of different colors, viewers can selectively track a subset of dots of a particular color by ‘paying attention’ to anything with this color. This is accomplished by activating neurons that are more sensitive to the corresponding feature wherever it appears in the display (Franconeri, 2013; Sàenz et al., 2003). Objects can also be selected by other features such as shape (Bertin, 2011). In a study where participants were asked to remember the colors of circles and squares, if they were probed that squares were more likely to be tested, their accuracy of reporting colors of squares was higher than that for circles, showing that shape-based attention can allow objects with that shape to be selected for encoding in working memory (Dube et al., 2017). Another study (Blaser et al., 2000) showed that people can track overlapping features in the same spatial region. In this study, participants were able to track one set of bars oriented in the same way overlaid with a second set oriented in a different way. For objects selected by motion features such as motion direction, it is possible to select and track one group of objects sharing the same motion direction among multiple groups with different motion directions (Levinthal & Franconeri, 2011).

The capacity limit of tracking features depends on which features are tracked. Some features, such as color, are stronger selection cues than others (Gleicher et al., 2013). The capacity limit also depends on whether the features are from the same or different feature categories. When tracking multiple features from the same category (e.g., red and yellow objects), tracking performance declines as compared to tracking only one feature (Cavanagh & Alvarez, 2005; Liu & Jigo, 2017). When tracking features from different categories (e.g., yellow objects and square-shaped objects, which is one color and one shape), tracking becomes easier. For example, in a display of multiple colors and multiple shapes, it is easy to track red circles, which is a combination of the color feature red and the shape feature circle (Huang & Pashler, 2007). Overall, evidence suggests a capacity limit on feature tracking of 1–2 features at once.

### Suggested design techniques for tracking tasks

*Show history traces*. Because dynamic information disappears over time and visual memory is limited, viewers lose that information if not immediately noticed. One solution is to leave static traces of the dynamic information, such as having objects leave a trail along the path that they travel. The traces represent the absent information in working memory (Harrower, 2003) so reduces memory demands for viewers. History traces can also encode changes in object features over time, such as varying trail thickness for size changes and changing colors of trail sections for hue changes. If a shape changes to another shape, the outline of the previous shape can be placed somewhere along that trail. At the same time, this technique must be used with restraint, as excessive use of traces increases visual complexity, which can impair performance in visual tasks. For example, visual search performance becomes less accurate and less efficient in displays with higher visual complexity such as cluttered scenes and noisy backgrounds (Neider & Zelinsky, 2011; Wolfe et al., 2002). One example of the use of this technique is in DimpVis, a tool that enables exploration of changes in data over time by allowing viewers to see the full trajectory trace of a selected data point and to drag the selected data point along its trace. This technique is subjectively preferred by viewers and leads to significantly faster task completion than small multiples for tasks including retrieving values and making comparisons (Kondo & Collins, 2014).

*Highlight relevant objects.* Given limited perceptual capacity, drawing viewers’ attention to relevant objects (e.g., objects to be tracked) helps them focus on smaller sets that could be within their capacity. To do so, designers can increase the salience of the subset of targets using features such as color, shape, transient flicker, or annotation (Munzner, 2014). In live presentations, language and gestures can also guide viewers’ attention toward the relevant objects and patterns of change at each stage while the presenter explains the animation, as demonstrated in Rosling’s TED talk.

*Move objects in stages*. To mitigate limits in tracking tasks, designers can move objects in stages, instead of simultaneously. One method suggested for this task is *item staging*, where objects move in subsets at a time, instead of all at once. For example, an animated visualization can have a subset of non-targets move first, and then have the subset of targets move after the movements of non-targets are completed, so the two subsets are more easily differentiated as single groups. However, item staging could lead to a loss of common motion information about whether objects in separate groups move in similar paths and makes it more difficult to predict when objects might begin to move (Chevalier et al., 2014).

*Incorporate interactivity*. Because viewers might not track all the relevant objects during their first view of an animation, interactivity enables re-inspection by allowing replay of portions of the animation, different (e.g., slower) speeds, and selective focus. This offers advantages over looping animations, which is a common approach used in data GIFs to show the visualization repetitively, especially when the number of frames is small. Looping often lacks clear markings of the start and end of the sequence and some technologies (e.g., GIF) do not provide speed control or pauses (Shu et al., 2020). Besides allowing viewer-controlled re-inspection, interactivity allows viewers to change the display’s design as if implementing the other techniques on their own according to the expected task. For example, interactivity can allow viewers to reduce the number of presented objects by filtering data, choose a subset of objects to have a different color (highlight relevant objects), show trails behind objects to indicate their previous motion trajectory (show history traces), and move only subsets of data points at a time (move objects in stages).

### Holistic judgments

Holistic judgments are often made when viewers *see changes to distribution and pattern*. For example, this task is performed to see the overall changes of a distribution flattening or data range increasing.

Broadly, holistic judgments are made in two ways:

#### Extracting average features (high capacity limit)

When viewers extract the average of a feature (such as the average size), they can accurately judge the average for a set of objects, regardless of the number of objects in this set, in strong contrast to the limits presented by the tracking tasks. For example, viewers could extract changes in average features such as dots becoming redder (color), bars growing (size), the number of objects increasing (numerosity), etc. Viewers can judge the average changes in the features across the group as a whole, even without being aware of the precise features of any individual member (Szafir et al., 2016). These features also include motion features including speed and direction. People can instantly compute an average motion direction (Watamaniuk et al., 1989; Williams & Sekuler, 1984) and can discriminate the average motion speed for a set of objects comprising many speeds with equal precision as for a set that contains a single speed (Watamaniuk & Duchon, 1992).

**Fig. 3:**
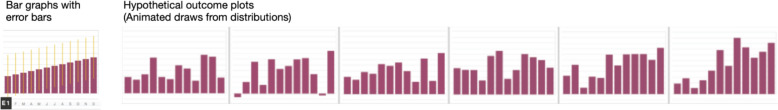
Example of extracting average features in animated data visualization. *Note*. A static error range graph versus hypothetical outcome plots (HOPs). The static visualization conveying uncertainty shows a single frame with ranges indicated by error bars, while animated HOPs display multiple frames with a possible dataset in each. An example of HOPs is shown in the New York Times (Irwin & Quealy, 2014).

People can accurately summarize average statistics for sets of objects shown simultaneously in space, or even shown sequentially over time (Szafir et al., 2016). This ability can help people understand statistical data distribution and uncertainty. For example, animated hypothetical outcome plots (HOPs) communicate uncertainty by showing a sequence of possible datasets sampled from a distribution (Hullman et al., 2015). The visual system allows viewers to quickly and accurately extract mental representations and find out the trend from such a sequence akin to a flipbook without requiring explicit memory. When asked to infer the underlying trend in either HOPs or static uncertainty graphs (Fig. 3), viewers were able to accurately infer this trend when presented as HOPs but not when presented as static error bars (Kale et al., 2019).

A special case of extracting average motion features involves optic flow, which arises when all objects or a subset have a uniform pattern of motion, including linear translation, expansion, retraction, rotation, and zooming in or out. An example in animated visualization is zooming into a smaller section of a network diagram. Optic flow refers to the global motion pattern generated when we move through an environment (Warren, 2008). These motion patterns provide perceptual cues of self-motion in our everyday activities (Crowell et al., 1999; Lappe et al., 1999). For example, forward walking generates a pattern of zooming in and turning around produces rotational motion. This motion perception system is powerful because it is subserved by a massive parallel processing system tuned to three primary axes that encode the dimensions of the visual field, and respond most strongly when the overall motion is equivalent to a viewer rotating about or translating along one of the axes (Nakayama, 1985), providing a parallel processing system that allows excellent holistic sensitivity to coherent optic flow motion.

While viewers can extract average feature information from large collections of objects, it is still beneficial for viewers to ‘pay attention’ to that collection, as opposed to another set of objects or another spatial area of a display. One study asked participants to compare between two target matrices each placed at the center of a display, which was also surrounded by an ensemble of circles of different sizes. Without being probed to pay attention to the surrounding ensemble, which should lead participants to focus spatial attention to only the display’s center, participants failed to detect changes in its average size. But when probed to pay attention to the surrounding ensemble, reports of the changes to the ensemble significantly improved in accuracy (Kimchi & Sabary, 2025). This suggests that, if animated designs intend for viewers to extract feature information from a large collection of objects, those animations should not require a simultaneous task that requires processing another set of objects or information from another location.

#### Evaluating changes to the shape envelope of a set of objects (limit unknown)

Another form of holistic judgment involves seeing sets of objects as a shape envelope (Franconeri, 2021). The shape envelope can be formalized as the convex hull, the smallest polygon enclosing all objects and all straight lines that connect any two objects, with objects as vertices. Some simple examples are three dots in a scatterplot forming a triangle or two bars in a barchart forming a trapezoid (Katzin, 2018; Mandler & Shebo, 1982). When objects move, their enclosing shape envelope changes accordingly. For example, in a dynamic histogram, the skewing, flattening, or rising of the distribution is likely perceived through the transformation of the overall shape of all of the bars, instead of through individual bar heights. Research further suggests that reporting the number of objects from shape envelopes that are canonical shapes requires less reaction time than from random shapes when there are more than four objects, exceeding the ‘subitizing’ range in which people can rapidly tell the accurate number without counting (Mandler & Shebo, 1982). However, current evidence provides little insight into the capacity limits of shape envelope perception. For example, it is not yet known how viewers evaluate changes to multiple shape envelopes at once, and how spatial separation influences whether objects are considered to be in the same shape envelope.

### Suggested design techniques for holistic judgments

The two holistic judgment tasks show relatively high capacity limits, so design techniques offer limited additional benefit. ‘Highlighting relevant objects’ would be relevant only if viewers should make holistic judgements about a subset of objects, by helping segment those objects for isolated processing. Similarly, ‘moving objects in stages’ may impair holistic judgments of the holistic motion information of the superset of objects, as the holistic motion perception could be lost when objects move asynchronously (Chevalier et al., 2014). For example, one study showed that when comparing mean, variance, and outlier across lines that were serially drawn through animation, participants were more accurate when the lines were presented synchronously than sequentially (Hu et al., 2024).

*Show history traces*. When viewers evaluate the average motion features of data objects, history traces can support this task by marking the motion trajectories for all objects so viewers can rely on the visible traces to extract the average direction and understand the overall pattern. When viewers evaluate the changes to the overall shape envelope of objects, history traces can facilitate this task by marking the shape outlines across time frames so viewers can compare across different visible shapes.

*Incorporate interactivity*. Interactivity allows viewers to replay the animated visualization to further engage with the holistic patterns. Designers can also enable showing history traces through an interactive control, showing the trajectory patterns or shape envelopes when viewers desire to. One benefit of combining interactivity with history traces is to avoid overcrowding—while showing the traces for all objects could aid identifying the overall pattern, too many traces on the screen could potentially become overwhelming, and only displaying the traces through interactivity could better align with the viewers’ need and preference.

### Noticing Objects Added or Removed (limit unknown, but likely low in busy displays)

Viewers often need to notice objects added or removed in animated displays to *recognize when data is filtered based on certain criteria*, when data are included where it was not present at the outset or removed from the existing display. This can occur by either 1) keeping the axes fixed while adding or removing data objects or 2) increasing or decreasing the scope of the display area to expand or contract the visible dataset. Viewers rely on seeing the general increase or decrease in the number of data objects to understand how the data are processed and presented by the designer. Viewers can also notice objects added or removed when they *understand data exceeding/entering the display area*. More specifically speaking, data objects that encode values exceeding the range of the display area travel out of the display, or data objects that encode the not yet included values travel into the display. Viewers rely on seeing new or old data objects to understand how the represented data values are changing.

**Fig. 4:**
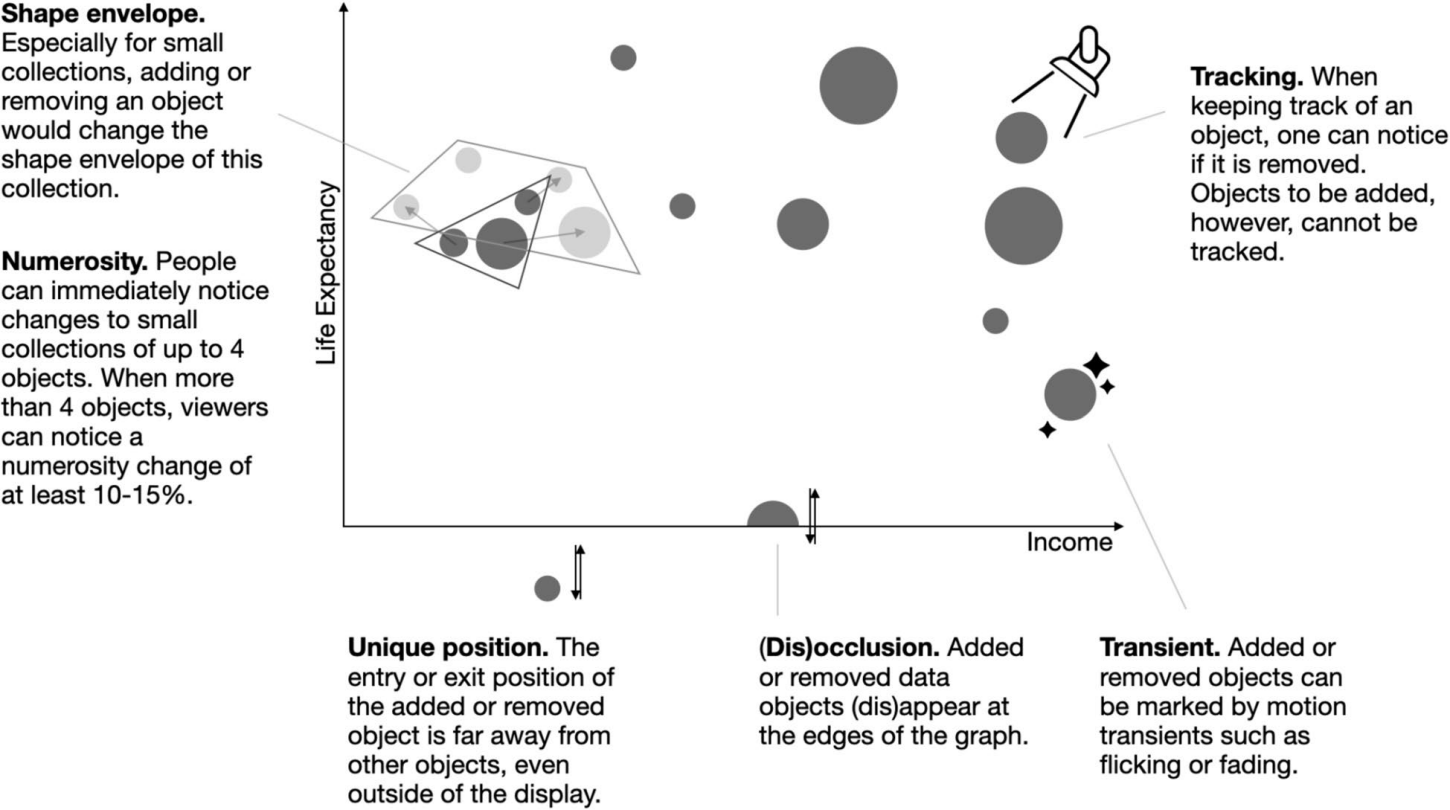
Summary of heuristics viewers might use to notice added or removed objects

Viewers can reliably notice removed objects when those objects are being actively tracked (although objects to be added cannot be tracked before they actually appear). In many cases, however, additions or removals are easily missed. When more than four objects are present, viewers can only keep track of a subset, and so are less likely to notice if an untracked object is removed. While earlier work suggested that new objects per se capture our attention (Yantis & Jonides, 1996), later work suggested that it is not the appearance of the new object itself that draws attention, but the transient signals (transient motion, looming, brightness change, etc.) that tend to accompany those events (Franconeri et al., 2005). Therefore, in most cases, people notice addition and removal via a set of heuristics (Most et al., 2005). We summarized the heuristics viewers might use to notice added or removed objects below and in Fig. 4.

One heuristic to notice the addition or removal of objects might be to notice changes in the number of objects. People can easily count small sets of up to four objects (Choo & Franconeri, 2014). For larger sets, viewers can still detect a change in number when the difference reaches about 10–15% of the total, even under dual-task conditions such as identifying a colored pattern in the same display (Burr et al., 2010). For example, on the whole, viewers can notice an increase in numerosity from 20 to 25 objects, but not from 20 to 21.

Another heuristic to notice added or removed objects might be noticing changes to the shape envelope of a set (e.g., a display changing from a triangle to a square might signal a new object). This is effective in small collections where each object forms a vertex of the shape envelope, so that even one added or removed object alters the shape envelope. In larger sets, however, many objects fall within the envelope rather than forming vertices, and additions or removal of such objects may not alter the shape envelope. Moreover, objects within the shape envelope could move to become vertices, changing the shape envelope without an actual addition.

As another potential heuristic to notice addition or removal, the object can have motion transients of short and swift changes, such as added objects flickering or fading in, while removed objects flickering or fading out. Viewers can efficiently find a flickering target among smoothly moving distractor objects (Pinto et al., 2008). Even slow flickers can capture attention as long as their frequency difference from distractors exceeds an absolute value rate of 5 Hz, with larger differences yielding faster detection (Cass et al., 2011). Accompanying an object’s appearance or disappearance with a brief change in brightness, opacity, or size can also improve detection of added or removed objects (Scarr et al., 2015).

Another heuristic might be the occlusion or disocclusion cue present when an object leaves or enters at the edges of the display. The object’s shape is cut off by the edge of the display and therefore looks different from ‘whole’ objects within the display (e.g., a half circle instead of a full circle). It also includes a motion transient as objects gradually become blocked or unblocked by the edges of the display. But existing evidence suggests that it is difficult for viewers to notice these different shapes, because the visual system broadly and automatically ‘completes’ occluded shapes. For example, finding an occluded square among complete squares in a crowded display is sluggish (Alexander and Zelinsky 2018; Rensink & Enns, 1998). In another study, for two incomplete shapes with one occluded but the other not (e.g., a half circle that abuts a square versus a half circle separate from a square), viewers took more time to search for the occluded circle than to search for the separate circle (Rauschenberger & Yantis, 2001). Occlusion and disocclusion in animated data visualizations might be difficult to notice, especially that viewers might be prioritizing other tasks such as tracking or extracting distributions. Viewers often fail to notice an unexpected appearance of a new object when tasked with counting the number of times existing objects bounce on the edges of the display (Most et al., 2005). However, if a tracked object that moves unpredictably in a display becomes briefly but completely occluded, tracking performance (MOT) is not impaired, and viewers can easily notice the disappearance and reappearance of the tracked object (Scholl & Pylyshyn, 1999).

Finally, another heuristic to notice an object leaving or entering a display might be the distance of the entry or exit position relative to other objects. Viewers can detect and identify outliers more easily when they are far away from other objects (Wang et al., 2019). In a graph, outlying positions are often close to the edges of a display, or even outside of the display. For objects traveling outside of the display, as compared to occlusion or disocclusion where objects are blocked at the edges, they exist on the screen for a longer time and travel for a longer distance before entering or leaving the display, or travel faster if the animation time is fixed.

### Suggested design techniques for noticing objects added or removed

*Show history traces*. When an object travels into or out of the display, showing its history trace can indicate its entering or leaving trajectory. For example, an object’s history trace intersecting with the edge of the display suggests that the object moved in or moved out.

*Highlight relevant objects*. Objects to be added or removed can be highlighted by leveraging a heuristic described above, motion transients, such as flickering or fading in/out. It captures the viewer’s attention to the changes to that object.

*Move objects in stages*. Designers can have objects added or removed as a separate rather than simultaneous step from the other motion ongoing in the animation. With *task staging*, an animation shows certain *types* of changes sequentially (Fisher, 2010). For example, in a display of a node-link network, viewers were faster and more accurate at judging how edges or nodes in that network disappeared or appeared when those two *types* of changes happened sequentially rather than simultaneously (Crnovrsanin et al., 2021).

*Incorporate interactivity*. With interactivity, viewers can replay an animated visualization to identify and observe the moment when there are objects added or removed, or slow down the animation speed so viewers can examine the changes more closely.

## Design guidelines

So far, we have reviewed the visual tasks needed to understand animated data visualizations, their perceptual limitations of those tasks, as well as the design techniques to help overcome those limits. To make this information most understandable, accessible, and usable for designers, we summarized an interactive webpage of guidelines. This is a living document: the implementation of the webpage will be continuous, and future updates will incorporate further information informed by both ongoing empirical research (see Sect. “Discussion and future work”) and interview feedback from users of the guidelines.

**Fig. 5:**
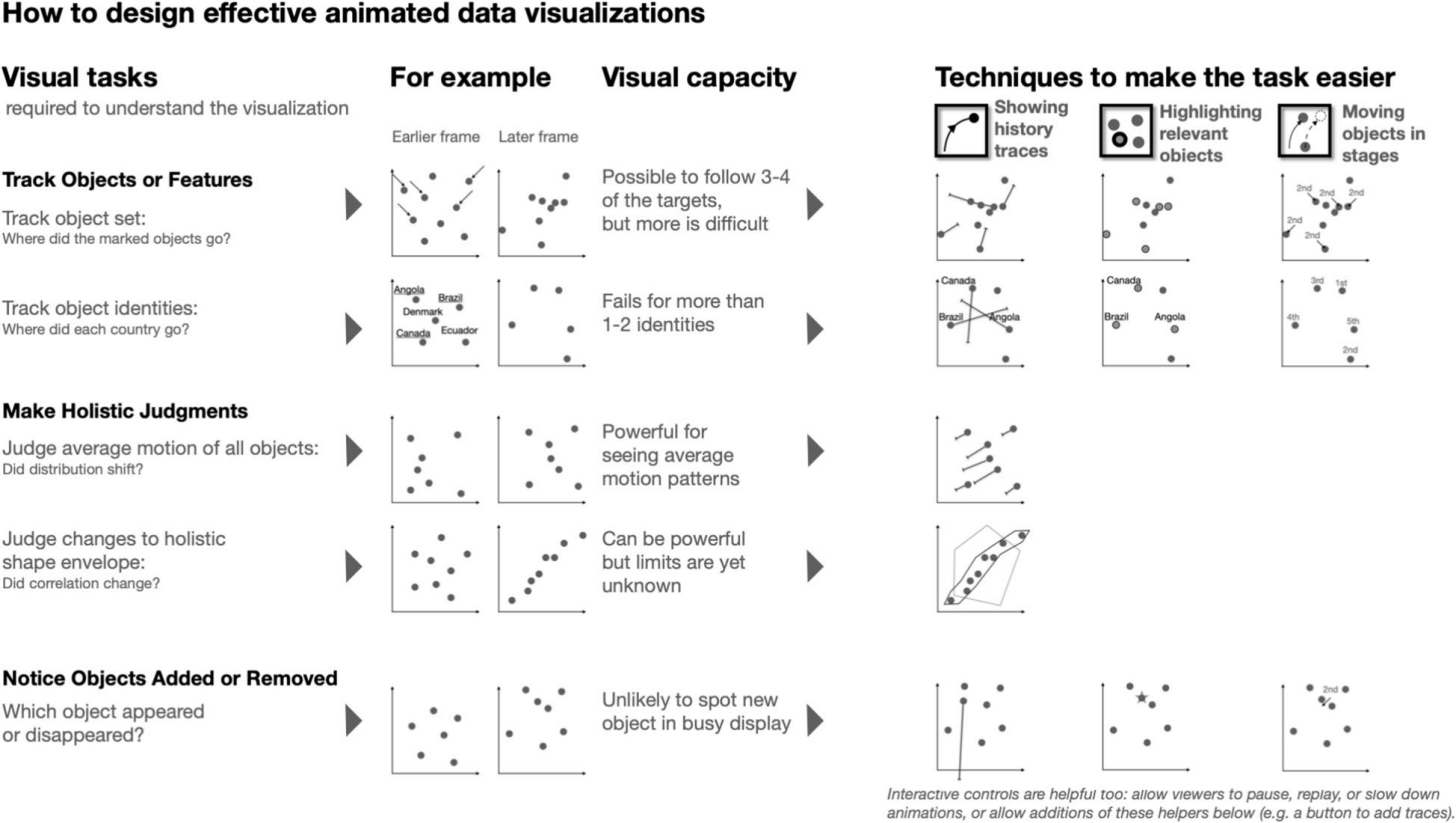
Preview of the homepage of the guidelines webpage. *Note*. The webpage of ‘How to design effective animated data visualizations’ is interactive so users can click on each visual task to read descriptions, see examples, and play demo videos

The home page of the guidelines (Fig. 5) presents a table of suggested techniques aligned with each visual task (described below). By clicking on any visual task, users are directed to video demos illustrating how displays can respect each task’s capacity limits. For example, one demo shows how tracking objects as a set of no more than four targets—and minimizing interference by other distracting objects—avoids exceeding viewers’ capacity limit. Additionally, each task also links to real-world examples of the application of design techniques.

To simplify the guidelines, we merged ‘tracking objects as individuals’ and ‘tracking features’ into one task ‘tracking object identities’ (capacity limit of 1–2 identities), given their shared limitations, even though their underlying visual mechanisms likely differ. This task, along with ‘tracking object set’ and ‘noticing objects added or removed,’ reflects restricted perceptual capacity limits. In these cases, performance can be improved by implementing one or more of the three design techniques, including leaving trails behind the targets, highlighting targets with a salient feature such as color or flickering, or moving targets as a separate group from distractors.

Interactivity is not included in the same way as the other suggested techniques in the guidelines as it is a cross-cutting strategy. It can enhance comprehension by allowing viewers to play, pause, and control the playback speed, or to activate any of the other three techniques, such as letting viewers click on certain objects to highlight them, or letting them decide when to move on to the next stage of the animation.

The design techniques we outlined are currently mapped to individual visual tasks. However, real-world animated data visualization might require viewers to perform multiple visual tasks simultaneously. In such cases, designers need to consider whether techniques applied to one task may interfere with another. For example, if history traces are used for two different tasks, viewers might confuse the two sets of traces unless clearly distinguished. Designers might also need to prioritize techniques for tasks with lower capacity limits. Even for single visual tasks, there are caveats for using techniques (such as the feature used to highlight relevant objects is not used for other encodings) and the relative effectiveness of techniques differ across tasks. These interactions and trade-offs require further empirical study (see Sect. “Discussion and future work”) before they can be incorporated into the guidelines. As noted earlier, refinding and updating the guidelines will be an ongoing process.

Consistent with its own design advice, the guideline webpage is interactive, that viewers of this webpage have control over what to see first and when to see it. Animations are played only when selected by the user, ensuring they play only when viewers are attending to them, thereby reducing cognitive load and preventing distraction from multiple simultaneous animations.

## Discussion and future work

We developed and presented guidelines that incorporate perceptual capacities and limitations into the design of animated data visualizations, supporting a range of visual tasks. They offer a structured means of applying established and developing knowledge on the perceptual limits to animated data visualization design in order to address known challenges experienced by viewers. The tasks and techniques proposed here are based on an iterative process combining work from multiple interdisciplinary literatures. While we are confident that they should help designers identify and resolve capacity limits within animated visualizations, that is a prediction that must be tested in future work. For example, a follow-up study could ask one group of designers to recreate one animated visualization with the guidelines as reference and another group to recreate the same animated visualization without the guidelines. The original and revision could then be presented to viewers for qualitative feedback and more formal experiments based on operationalized goals for each visualization.

In creating these guidelines, we identified several areas where empirical work remains limited, particularly at the intersections of tasks, limits, and design techniques. We are addressing these gaps in ongoing work that could augment our guidelines with additional evidence. For example, with respect to the task of ‘noticing objects added or removed,’ we are testing whether objects are more readily noticed if they appear or disappear via unique positions rather than through occlusion or disocclusion. Another line of research examines the perceptual limits of multiple visual tasks when they occur concurrently, such as evaluating changes in average features while tracking object identities. In addition, while our work emphasizes strategies for improving animated data visualizations, in some situations, static visualization may lead to better interpretation and should be used instead of animated ones. Identifying and articulating these situations, and incorporating them into the guidelines, represents an important direction for expanding the guidelines’ practical relevance.

While the present guidelines focus on the perceptual limits for processing animated visualizations, those visualizations also face challenges beyond raw processing capacity. For example, designers should consider a viewer’s prior knowledge and expertise that would allow them to appropriately deploy their limited perceptual resources. In one experiment, students learned quadratic equations with either static graphs (a graph with labels of major steps for transformation between two lines of different equations) or animated graphs (direct transformation between two lines of different equations). Students with more prior knowledge in math performed better with the animated graph than students with less prior knowledge in math. Students with more prior knowledge also performed better with the animated graph than with the static graph, because their sufficient knowledge base equipped them to deal with potential issues relevant to animations such as transient information and limited working memory capacity (Kalyuga, 2008).

We hope that the interactive guideline webpage will serve as a practical resource for designers seeking to enhance their data visualizations with animation. Future work should assess the effectiveness of the guidelines in terms of their utility, memorability, and influence on design practice. Key questions include: do the guidelines clearly communicate the tasks, their limits, and techniques for overcoming them? And crucially, do visualizations designed according to these guidelines indeed result in improved interpretability for viewers?

## Data Availability

Collected examples of real-world animated data visualizations and analyses.
